# Landscape Genomics Provides Evidence of Ecotypic Adaptation and a Barrier to Gene Flow at Treeline for the Arctic Foundation Species *Eriophorum vaginatum*

**DOI:** 10.3389/fpls.2022.860439

**Published:** 2022-03-24

**Authors:** Elizabeth Stunz, Ned Fetcher, Philip Lavretsky, Jonathon E. Mohl, Jianwu Tang, Michael L. Moody

**Affiliations:** ^1^Department of Biological Sciences, University of Texas at El Paso, El Paso, TX, United States; ^2^Institute for Environmental Science and Sustainability, Wilkes University, Wilkes-Barre, PA, United States; ^3^Department of Mathematical Sciences, Border Biomedical Research Center, University of Texas at El Paso, El Paso, TX, United States; ^4^Marine Biological Laboratory, The Ecosystems Center, Woods Hole, MA, United States

**Keywords:** arctic, climate change, *Eriophorum vaginatum*, landscape genomics, environmental niche modeling, genotype-environment association analyses, refugia

## Abstract

Global climate change has resulted in geographic range shifts of flora and fauna at a global scale. Extreme environments, like the Arctic, are seeing some of the most pronounced changes. This region covers 14% of the Earth’s land area, and while many arctic species are widespread, understanding ecotypic variation at the genomic level will be important for elucidating how range shifts will affect ecological processes. Tussock cottongrass (*Eriophorum vaginatum* L.) is a foundation species of the moist acidic tundra, whose potential decline due to competition from shrubs may affect ecosystem stability in the Arctic. We used double-digest Restriction Site-Associated DNA sequencing to identify genomic variation in 273 individuals of *E. vaginatum* from 17 sites along a latitudinal gradient in north central Alaska. These sites have been part of 30 + years of ecological research and are inclusive of a region that was part of the Beringian refugium. The data analyses included genomic population structure, demographic models, and genotype by environment association. Genome-wide SNP investigation revealed environmentally associated variation and population structure across the sampled range of *E. vaginatum*, including a genetic break between populations north and south of treeline. This structure is likely the result of subrefugial isolation, contemporary isolation by resistance, and adaptation. Forty-five candidate loci were identified with genotype-environment association (GEA) analyses, with most identified genes related to abiotic stress. Our results support a hypothesis of limited gene flow based on spatial and environmental factors for *E. vaginatum*, which in combination with life history traits could limit range expansion of southern ecotypes northward as the tundra warms. This has implications for lower competitive attributes of northern plants of this foundation species likely resulting in changes in ecosystem productivity.

## Introduction

Investigating the adaptive constraints of plants is critical to better determine how different species and communities may respond to an altered climate (Shaver et al., 2000) and how best to predict ecological community shifts in the future (Ikeda et al., 2017b; Smith et al., 2019). Ecological communities are transforming and species distributions are shifting in response to a changing climate. Such changes are more evident in environments with a steep transition between ecosystems, or in biomes experiencing extreme effects of global climate change (Chen et al., 2011; Felde et al., 2012). Among these, arctic and alpine environments are areas experiencing some of the most pronounced effects of climate change, with a temperature increase of more than 0.5°C per decade over the past 40 years in the arctic tundra alone (Serreze et al., 2000; Sturm et al., 2005; Chandler et al., 2015) and an 11°C increase projected by 2100 (IPCC, 2014). Changes in climate have already led to plant community shifts (Villarreal et al., 2012; Hollister et al., 2015), including northerly shifts in treeline (Harsch et al., 2009) and increased shrub density (Tape et al., 2006). These shifts are also important for foundation species in which ecotypic variation influences gross primary productivity (GPP) and ecosystem response to climate change (Curasi et al., 2019). This has implications for the carbon cycle, as the soil and vegetation in the Arctic store large amounts of carbon (Schaefer et al., 2014). Determining population structure, or lack thereof, across species’ range distributions and understanding whether environmental variables are responsible for such patterns are both essential to model potential responses to ongoing climate change (Smith et al., 2019; Reiskind et al., 2021).

Among geological events with lasting effects on populations, glaciation events often serve as a major factor leading to genetic divergence (Ferris et al., 1993; Soltis et al., 1997). In fact, comparative analyses of spatial genetic structure and diversity of multiple circumpolar arctic plant species identify Pleistocene glaciation, the resulting physical barriers, and glacial refugia as responsible for shaping patterns across the Arctic (Alsos et al., 2012; Eidesen et al., 2013). The Beringian region, which remained mostly unglaciated throughout the Pleistocene glacial-interglacial cycles, is known to have served as an important refugium for multiple arctic plant and animal species, and therefore served as sources for post-glacial expansions of these populations (Abbott and Brochmann, 2003; Alsos et al., 2005; Brubaker et al., 2005). Identifying refugium origin for the contemporary distribution of arctic taxa has been an emphasis of circumpolar population genetic studies (Alsos et al., 2005, 2007), whereas evidence for differently adapted genotypes advancing from refugia has only begun to be explored (Ikeda and Setoguchi, 2017; Napier et al., 2019; Wang et al., 2021).

Refugia like the Beringian region had a heterogeneous landscape (Billings, 1992; Lapointe et al., 2017), and local adaptations within these landscapes could have a comparably strong effect on the post-glaciation distribution of genotypes. During the Last Glacial Maximum [LGM; ∼20 thousand years before present (kyr BP)], tundra-steppe in the north and spruce woodlands in the south of the Beringian refugium were isolated by glaciation over the Brooks Range (Billings, 1992; Lapointe et al., 2017), forming potential subrefugia. Post-glaciation, these habitats are largely differentiated by treeline extending along the interface of the taiga-tundra biomes, which functions as an ecological barrier (Chapin et al., 1996) generally delimited by permafrost depth, soil availability, growing season temperature, and reduced albedo (Billings, 1973; Chapin et al., 1996). Treeline can also pose a physical barrier for wind-pollinated or wind-dispersed plants, both of which are dominant among arctic taxa (Dahl, 1963; Chapin et al., 1996). Therefore, there is both older as well as more contemporaneous landscape heterogeneity in the Arctic that could lead to adaptive and physical barriers through time.

Landscape-scale genetic constraints for arctic plant species have been proposed, especially for ecotypic specialization and adaptational lag (Bennington et al., 2012; Mazer et al., 2013; McGraw et al., 2015). Local adaptation across a species’ range can lead to differences in the thermal optima or climatic niches of populations, resulting in ecotypes with narrower environmental tolerances if adaptation is strong (Forester et al., 2016; Peterson et al., 2018). This is pertinent to the Alaskan Arctic where there has been an environmental cline that has remained relatively stable over the last ∼6,000 years (Billings, 1992). Lag in dispersal and establishment can hamper plant ecotypes from adjusting their ranges to track and remain in climate optima, resulting in reduced fitness of local genotypes, or adaptational lag. Here we use tussock cottongrass (*Eriophorum vaginatum* L.; Cyperaceae), a sedge that exemplifies arctic plant distribution across tundra and taiga biomes and occurs throughout the Beringian region, to investigate landscape genomic patterns.

*Eriophorum vaginatum* is a wind-pollinated and wind-dispersed sedge, and a foundation species of the moist acidic tundra (MAT). It has a circumarctic and circumboreal distribution and is a common component of taiga forest in muskeg and bogs (Wein, 1973; Fetcher and Shaver, 1982, 1983; Brown and Kreig, 1983). As a foundation species, *E. vaginatum* plays a strong role in structuring the ecological network where it occurs. In tundra sites, *E. vaginatum* can account for up to one-third of ecosystem productivity (Chapin and Shaver, 1985), and is prevalent throughout Alaska, northern Canada, and northern Russia. Because tussocks are densely distributed and can persist over 100 years, new recruitment is likely uncommon (McGraw and Shaver, 1982; Gartner et al., 1983). This is pertinent under climate change, as the climate optima for ecotypes of tussock cottongrass was displaced ∼140 km northwards between 1993 and 2010 in Alaska (McGraw et al., 2015).

**FIGURE 1:**
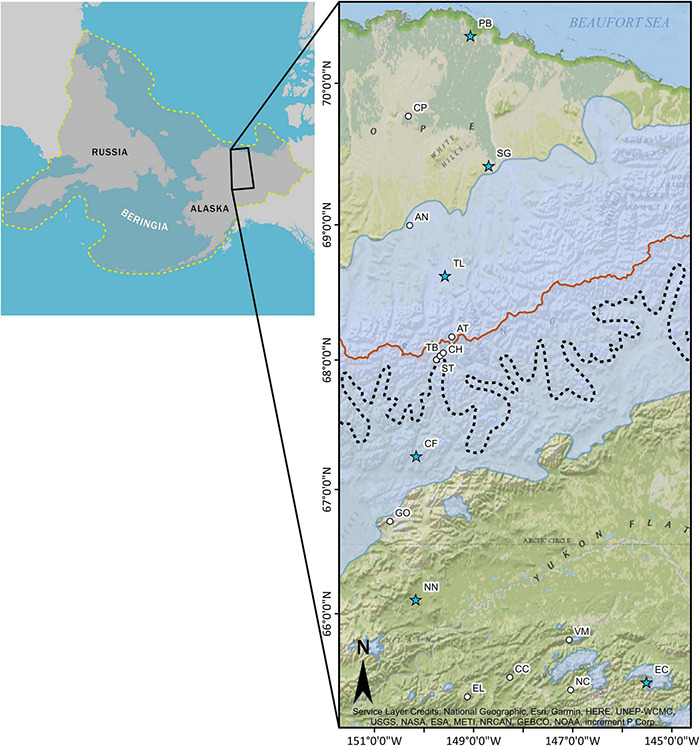
Map of *Eriophorum vaginatum* sampling locations and modeled maximum Pleistocene glacial extent (Kaufman and Manley, 2004; Kaufman et al., 2011) along a latitudinal gradient in north central Alaska. Blue stars designate reciprocal transplant gardens (Shaver et al., 1986; Bennington et al., 2012). Treeline is indicated by the dashed black line and the Continental Divide is indicated by the burnt orange line. The inset shows the extent of the Beringian region, outlined with a dashed yellow line. The 17 collection site abbreviations are as for Table 1.

Importantly, over 30 years of reciprocal transplant studies have uncovered measurable phenotypic variation of *E. vaginatum* across an arctic latitudinal gradient within the geographic range of the eastern Beringian refugium (Figure 1). These include: (1) Tussocks transplanted back into their home sites showing home site advantage in flowering rates and survival; (2) Some tussock adaptations correlated with latitude of population origin in light-saturated photosynthetic rate and stomatal density; and (3) Tussock adaptations related to leaf phenology and plastic responses correlated with the site of origin occurring north or south of the treeline, which signifies the arctic tundra/taiga ecosystem shift (Fetcher and Shaver, 1990; Bennington et al., 2012; Peterson et al., 2012; Souther et al., 2014; Parker et al., 2017, 2021). These adaptations have led to the hypothesis that across this latitudinal gradient there are genetic constraints related to ecotype site of origin, and ecological adaptations should be present at multiple scales that will be manifest through landscape genomic analyses.

Here we use thousands of double-digest Restriction Site-Associated DNA sequencing (ddRAD-seq) single nucleotide polymorphisms (SNPs) to investigate broad patterns of population structure, gene flow, and associations with landscape variables in *E. vaginatum* populations along the latitudinal gradient in the north central Alaskan Arctic that is the site of the long-term ecological studies cited above. We address the following questions: (1) Is population structure delimited at the ecosystem level among these populations? (2) Is there evidence of genetic structure linked to adaptation for latitude of origin? We hypothesize that the Brooks Range glaciation within the Beringian refugium effectively isolated populations to the north and south and that this resulted in the confinement of *E. vaginatum* to putative subrefugia as glaciers expanded before the LGM. When separated for a sufficient amount of time, genetic drift and/or divergent selection should have altered the genomes of the isolated *E. vaginatum* populations, and population structure analyses should demarcate these isolated populations as unique. More recently, treeline may be expected to pose an important barrier to gene flow for contemporary populations of *E. vaginatum*, as the treeline ecotone represents the current boundary between glacially isolated habitats. Finally, we hypothesize that genetic correlation associated with environmental predictors will be uncovered along the stable arctic latitudinal cline corresponding to local niche dynamics that have resulted in the ecotypic variation identified through 30 + years of reciprocal transplant garden studies.

## Materials and Methods

### Study Area

The study area consisted of a latitudinal gradient covering ∼426 km in north central Alaska (Figure 1 and Table 1), from north of Fairbanks (65.433°, −145.512°) to Prudhoe Bay (70.327°, −149.065°). The Continental Divide at the crest of the Brooks Range divides the region into south slope and north slope components, roughly at the intersection of two climatic regions, the Arctic and the interior (Haugen, 1982; Brown and Kreig, 1983). Beginning just north of Fairbanks, taiga vegetation is dominant, with forest and lowland treeless bogs comprising much of the interior. Alpine or tussock tundra is found once elevations exceed ∼700 m. Treeline occurs on the south slope of the Brooks Range (Figure 1), while tundra plant communities are found north of treeline. Permafrost is continuous north of treeline and discontinuous in most of the interior, where soil parent materials, slope angle and aspect, drainage, and vegetation often indicate permafrost presence (Brown and Kreig, 1983). In general, the interior is warmer and annual rainfall declines north of the Brooks Range toward the coastal plain (Supplementary Table 1).

**TABLE 1:** Collection sites, GPS coordinates, elevation (meters), and vegetation type of *Eriophorum vaginatum* in north central Alaska.

Site	Latitude, Longitude	Elevation (m)	Vegetation type	Cluster
Eagle Creek (EC)	65.4332°, −145.5118°	771	MAT	EC
Nome Creek (NC)	65.3646°, −147.0406°	511	Muskeg	South
Victoria Mountain (VM)	65.7832°, −147.0681°	960	MAT	South
Colorado Creek (CC)	65.4705°, −148.2666°	192	Muskeg	South
Elliott Highway (EL)	65.3081°, −149.1230°	720	MAT	South
No Name Creek (NN)	66.1171°, −150.1676°	167	Tussock bog	South
Gobbler’s Knob (GO)	66.7459°, −150.6862°	520	Muskeg	South
Coldfoot (CF)	67.2631°, −150.1591°	321	Muskeg	South
South of Timberline (ST)	68.0006°, −149.7469°	702	MAT	South/North
Timberline (TB)	68.0300°, −149.6737°	760	MAT	North/South
Chandalar (CH)	68.0518°, −149.6115°	968	MAT	North
Atigun Camp (AT)	68.1730°, −149.4392°	1,063	MAT	North
Toolik Lake (TL)	68.6292°, −149.5778°	758	MAT	North
Anaktuvuk (AN)	68.9945°, −150.2871°	341	MAT	North
Sagwon (SG)	69.4244°, −148.6976°	299	MAT	North
Coastal Plain (CP)	69.7704°, −150.3128°	173	MAT	North
Prudhoe Bay (PB)	70.3270°, −149.0645°	8	MAT	North

*MAT, Moist Acidic Tundra. Genetic cluster assignment based on STRUCTURE analysis at K = 3 (Figure 4).*

### Sample Collection

**FIGURE 2:**
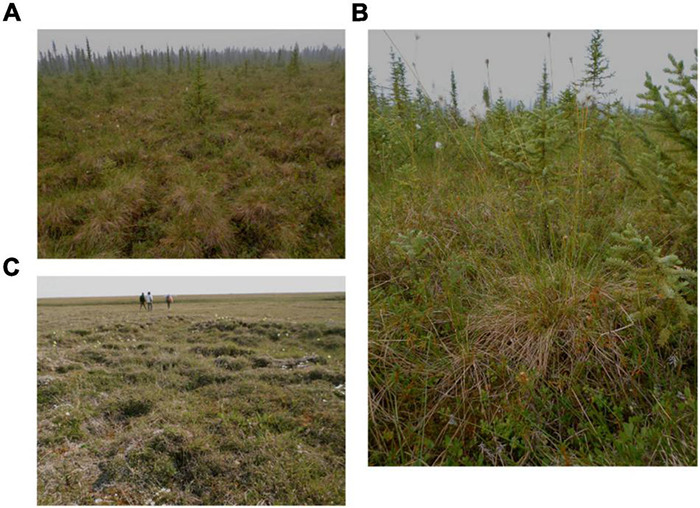
Images showing **(A)** the habitat of the Coldfoot sampling location south of treeline, **(B)** a mature *Eriophorum vaginatum* tussock at Coldfoot, and **(C)** the habitat of the Prudhoe Bay sampling location in north central Alaska. All photos by E. Stunz.

During the summers of 2015, 2016, and 2017, leaf samples were collected from *E. vaginatum* from 16–18 individuals at each of 17 sites (273 accessions) along the latitudinal gradient following the Dalton Highway (Figures 1, 2). We sampled away from reciprocal transplant gardens and used comparably large individuals to avoid the potential of using plants that would have germinated since the transplant gardens were established. Logistical access to this region is limited and cost prohibitive outside of the Dalton Highway as it can only be accessed by helicopter, which was needed for two coastal plain collections. Latitudinal distance between populations was ≤ 0.75° with denser sampling near treeline and at the southern end of the range. Individuals were sampled at least 50 m from the roadside and at least 10 m apart to minimize bias based on same seed parentage among the tussocks. Sampled leaves were dried and stored in silica gel. Sites north of treeline are classified as MAT (Britton, 1966; Wein and Bliss, 1974; Shaver et al., 1986), while most sites south of treeline are muskeg or tussock bog unless above 700 m (Table 1; Cleve and Dyrness, 1983; Kummerow, 1983; Shaver et al., 1986). The sampling strategy was designed to include sites that overlapped with gardens used in long-term ecological studies (Shaver et al., 1986).

### DNA Extraction and Double-Digest Restriction Site-Associated DNA-Seq Library Preparation

Genomic DNA was extracted from 50 mg of dried leaf tissue for 16 samples per site using a modified CTAB method (Doyle and Doyle, 1987). DNA concentrations were quantified using the Qubit dsDNA BR Assay Kit (Invitrogen, Waltham, MA, United States) and Qubit 3.0 Fluorometer (Thermo Fisher Scientific, Waltham, MA, United States). DNA samples with a minimum concentration of 0.02 μg/μL were included for preparation of double-digest Restriction Site-Associated DNA (ddRAD)-seq libraries, which generally followed DaCosta and Sorenson (2014) and Hernández et al. (2021).

To assemble ddRAD libraries, 43 μL of each ∼25 ng/μL DNA sample was digested with 20 U of *Eco*RI and 20 U of *Msp*I restriction enzymes (New England Biolabs, Ipswich, MA, United States; cut sites: 5′-GAATTC-3′; 5′ -CCGG-3′) and 5 μL of 10 × NEBuffer 4 (New England Biolabs, Ipswich, MA, United States) in a thermocycler at 37°C for 30 min followed by enzyme deactivation at 65°C for 20 min. Custom barcodes and indices were ligated to digested DNA, with the addition of 50 nM of custom *Eco*RI and *Msp*I adapters (containing individual barcodes), 2 μL of 10 × NEBuffer (New England Biolabs, Ipswich, MA, United States), 0.6 μL of rATP (Promega, Madison, WI, United States), 0.4 μL of ddH_2_O, and 1 μL of T4 DNA ligase (New England Biolabs, Ipswich, MA, United States) to each 50 μL sample of digested DNA. Ligation was completed in the thermocycler (22°C for 30 min), followed by enzyme deactivation (65°C for 20 min). Adapter-ligated DNA fragments were then double-side size selected with a 0.8 × SPRI bead clean up. Right-sided selection for large fragments was done with AMPure XP beads (Beckham Coulter, Inc., Brea, CA, United States) added at 0.55 × volume of the starting ligated DNA solution, and the supernatant transferred to new tubes. Subsequently, a left-sided size selection was done with 0.25 × volume AMPure XP beads added to the supernatant. The supernatant was then discarded and the beads were washed twice with 80% ethanol and air-dried. DNA was re-suspended with 25 μL ddH_2_O and eluted for a minimum of 30 min.

To amplify size-selected DNA fragments, PCR was performed with a solution of 15 μL template DNA, 30 μL of Phusion High-Fidelity PCR Master Mix (Thermo Fisher Scientific, Waltham, MA, United States), 9 μL of ddH_2_O and 3 μL of each 10 μM primer specific to sequences at the ends of each custom barcode and index (see DaCosta and Sorenson, 2014). PCR was performed in a thermocycler: 98°C for 30 s; 22 cycles at 98°C for 10 s, 60°C for 30 s, 72°C for 40 s and 72°C for 5 min. Amplified DNA fragments across samples were cleaned using a 1.8 × AMPure XP bead clean-up protocol (Beckham Coulter, Inc., Brea, CA, United States). After adding beads and discarding the supernatant, beads were washed twice with 80% ethanol and re-suspended in 40 μL ddH_2_O. Finally, cleaned PCR products were quantified with the Qubit dsDNA BR Assay Kit (Invitrogen, Waltham, MA, United States) and Qubit 3.0 Fluorometer (Thermo Fisher Scientific, Waltham, MA, United States). Additionally, samples were visualized with gel electrophoresis to ensure that all were similarly size-selected. Equimolar concentrations of samples with unique barcode combinations were pooled. Multiplexed libraries were sequenced at the Center for Genome Research and Biocomputing at Oregon State University on an Illumina HiSeq 3000.

### Single Nucleotide Polymorphism Identification and Genotyping

Files of raw Illumina read sequence data were de-multiplexed and analyzed via the *de novo* assembly method in STACKS 2.41 (Catchen et al., 2011, 2013), and using in-house Python scripts. First, the process_radtags program in STACKS was used to filter out reads with a Phred Quality score < 33, remove reads without intact radtags, and trim all reads to 145 bp. The sequences were then processed in the program ustacks to align the short-read sequences into “stacks.” Stacks were compared using a maximum likelihood framework (Hohenlohe et al., 2010) to identify loci and detect SNPs. The minimum depth of coverage for stack creation (m) was set to three (m 3; default), The maximum distance (in nucleotides) between each stack (M) was set to four (M 4) and all other parameters were set to default values. The cstacks program was used to build a catalog of consensus loci based on matching sets of reads built in ustacks. The number of mismatches permitted between sampled loci when building a catalog (n) was set to four (n 4). Parameter choice was guided by optimization methods of Paris et al. (2017) by assessing the number of loci and SNPs retained across 80% of individuals from each site (r80) for values 1–9 for M, assuming M = n, and fixing m to 3 (Paris et al., 2017; Rochette and Catchen, 2017).

Sets of putative loci were then compared to the catalog of loci with the sstacks program. Data was arranged by locus, instead of by sample, with the tsv2bam program. The ddRAD data was analyzed locus by locus across all individuals to genotype individuals for each SNP with the gstacks program. The data set was then pruned for minor alleles occurring in less than 1% of reads, as rare genotypes are likely the result of sequencing error (Tabangin et al., 2009). The STACKS populations program was run to retain only loci found in all sampled populations with ≤ 20% missing data. Two sets of SNPs were created: one single SNP per locus “stack” set containing putatively neutral markers (or the neutral dataset) and a set containing all SNPs (or the comprehensive set) used for RDA analysis.

Loci under balancing or directional selection were identified with BayeScan v.2.1 (Foll and Gaggiotti, 2008) via a Bayesian *F*_*ST*_ outlier test with default values, 20 pilot runs of 5,000 iterations were implemented, followed by 100,000 iterations, sampled every 10 iterations, with a 50,000 iteration burn-in. Outputted iterations were increased to ensure convergence of the MCMC chain (confirmed by visual assessment). In addition, prior odds settings were increased to 500 for the comprehensive dataset, as recommended for candidate loci identification in large datasets (Foll, 2010), and held at 100 for the neutral dataset. Candidate outlier loci were identified as those with a *q*-value < 0.05 [or a false discovery rate (FDR) of 5%]. Additionally, SNPs deviating from Hardy-Weinberg Equilibrium (HWE; *p* ≤ 0.001) were identified with PLINK^1^ v.1.07 (Purcell et al., 2007). Outlier loci and SNPs deviating from HWE were removed to create the neutral SNP data set used for estimating genetic diversity, demographic statistics, and population structure.

### Patterns of Genomic Diversity

Heterozygosity [observed (H_*o*_) and expected (H_*e*_)], allelic richness (Ar) and the inbreeding coefficient (*F*_*IS*_) were estimated across all loci and for each population with the *divBasic* function in the *diveRsity* R package v.1.9.90 (Keenan et al., 2013). Private alleles were estimated with the R *PopGenReport* package v.3.0.4 (Adamack and Gruber, 2014; Gruber and Adamack, 2015). Effective population size (*Ne*) was estimated with NeEstimator v.2.1 (Waples and Do, 2008; Do et al., 2014) using the bias-corrected linkage disequilibrium method (Waples, 2006), random mating, and an allele frequency threshold of ≥ 0.02 for *Ne*_*LD*_ calculation.

### Population Structure

Pairwise estimates of Weir and Cockerham (1984) unbiased *F*_*ST*_ were calculated between populations using GENODIVE 2.0b2.5 (Meirmans and Van Tienderen, 2004). The neutral dataset was processed in STRUCTURE v.2.3.4 (Pritchard et al., 2000; Falush et al., 2003), which uses a Bayesian clustering algorithm to assign individuals to genetic clusters (*K*). The analysis was comprised of 20,000 burn-in iterations followed by 50,000 replicates of each population value (*K* = 1–10), and each run was conducted 10 times (Schweizer et al., 2016). To determine the optimal value of *K*, the Δ*K* statistic (Evanno et al., 2005) was evaluated using STRUCTURE HARVESTER v.0.6.94 (Earl and VonHoldt, 2012). The *greedy* method and 1,000 random permutations were used in CLUMPP v.1.1.2 to account for variation in cluster assignment across STRUCTURE runs. Bar charts displaying the proportion of cluster membership for each individual were created and modified with DISTRUCT v.1.1 (Rosenberg, 2004). Population structure was also investigated using Discriminant Analysis of Principal Components (DAPC) and Bayesian clustering in the R package *adegenet* v.2.0.1 (Jombart et al., 2010). DAPC is useful to avoid *a priori* assignment and corroborate results of STRUCTURE as it provides a non-model-based method to estimate cluster assignment of individuals. The *find.clusters* function was run in *adegenet* to transform allele frequencies and determine the optimal number of clusters (*K* = 1–10) via *k*-means clustering of principal components and the Bayesian Information Criterion (BIC) (Jombart et al., 2010).

Hierarchical partitioning of genetic variance was evaluated with an analysis of molecular variance (AMOVA) (Excoffier et al., 1992) using the *poppr.amova* function with 99 permutations in the *poppr* R package v.2.8.0 (Kamvar et al., 2014, 2015). To determine gene flow patterns in our study area, the *divMigrate* function of the *diveRsity* R package v.1.9.90 (Keenan et al., 2013) was used to estimate asymmetric gene flow in relation to contemporary levels of genetic diversity between populations. Nei’s *G*_*ST*_ method was used to calculate values of relative directional migration between sampled sites and investigate source and sink dynamics. To test whether migration between populations was significantly asymmetrical, 1,000 bootstrap replicates were performed to calculate 95% confidence intervals (Sundqvist et al., 2016).

### Demographic and Environmental Niche Modeling

An environmental niche model (ENM) was used to create environmental suitability maps, to test various demographic models, and predict the geographical distribution of *E. vaginatum* suitable habitats past and present within the range of the Beringian refugium. To build an ENM, the species distribution was modeled for the present and projected into climate scenarios of the mid-Holocene (∼6 kyr BP), and the LGM (∼20 kyr BP). The initial ENM was built with 19 bioclimatic variables obtained from the WorldClim 2.0 Bioclimatic database (Fick and Hijmans, 2017) and derived from climatic records from 1970 to 2000 with the maximum entropy algorithm of MaxEnt v.3.4.3 (Phillips et al., 2006; Phillips and Dudík, 2008). MaxEnt uses species occurrence data and predictor variables to predict probability distribution of a species. WorldClim data were downloaded at 30 arc seconds spatial resolution. *Eriophorum vaginatum* species occurrence data were obtained from the Alaska Vegetation Plots Database (Nawrocki et al., 2020), adding 1,228 occurrences to the 17 sampled sites used in this study, for a total of 1,245 unique species occurrence records.

ENMEVAL v.2.0.2 (Muscarella et al., 2014) was used to determine optimal parameters, feature class (FC), and regularization multiplier (RM) settings for MaxEnt. A total of 64 models were created using eight FC combinations (L, LQ, LQP, H, T, LQH, LQHP, LQHPT in which L = linear, Q = quadratic, H = hinge, P = product and T = threshold) (Muscarella et al., 2014) and eight RMs (0.5, 1.0, 1.5, 2.0, 2.5, 3.0, 3.5, 4.0) (Manzoor et al., 2020). The “block” method was implemented to partition data into calibration and evaluation datasets in order to evaluate model performance (Muscarella et al., 2014; González-Serna et al., 2019). The model with the lowest Akaike Information Criterion corrected for small sample size (AICc; Burnham and Anderson, 2002) was identified as the optimal model and implemented for the final run in MaxEnt. To obtain a subset of uncorrelated environmental variables, a Pearson correlation coefficient cutoff (*r* ≥ 0.75 and *r* ≤ −0.75) was applied to remove variables for the final MaxEnt run (Dormann et al., 2013; Manzoor et al., 2020). Modeled LGM layers for retained environmental variables were derived from the Community Climate System Model (CCSM) (Braconnot et al., 2007) and included as a projection layer in MaxEnt. ARCMAP v.10.7.1 (ESRI, 2011) was used to classify cell values (with a continuous range of 0–1) of current and LGM rasters into 20 equal interval habitat suitability bins (González-Serna et al., 2019) to reduce file size and speed up processing. A Mid-Holocene climate raster was created by averaging current and LGM layer habitat suitability bin values. Respective output raster files for current environmental conditions were obtained for use in CIRCUITSCAPE (McRae, 2006).

To examine the landscape resistance effects of different vegetation classes and canopy cover on gene flow, 30 m categorical land cover data were obtained from the National Aeronautics and Space Administration Arctic-Boreal Vulnerability Experiment (NASA ABoVE) ABoVE: Landsat-derived Annual Dominant Land Cover Across ABoVE Core Domain, 1984–2014 dataset (Wang et al., 2019). The 15-class system used for this data was condensed into four categories: forest, woodland, shrubland, and other. Forest included Evergreen Forest, Deciduous Forest and Mixed Forest classes with woody vegetation > 3 m tall and > 60% canopy coverage. The woodland category also included vegetation > 3 m tall, but with 30–60% canopy coverage. Shrubland comprised Low Shrub, Tall Shrub and Open Shrub classes with woody vegetation between 5 cm and 3 m tall and with 30–60% canopy coverage. Other included Herbaceous, Tussock Tundra, Sparsely Vegetated, Fen, Bog, Shallow/littoral, Barren, and Water classes, in addition to areas with missing data, which are likely covered by snow or ice. The dataset included 31 raster bands of land cover data, each corresponding to a year during 1984–2014 of land cover classification. To determine and implement the most frequent classification during this time series, a per pixel summarization was performed across the bands. Using ARCMAP, the original 30 m resolution dataset was resampled to 60 m to decrease processing requirements during runs in CIRCUITSCAPE.

A series of models was designed to examine the effects of isolation by distance (IBD), resistance (IBR), and environment (IBE) on genetic distance and run in CIRCUITSCAPE following Van Strien et al. (2012) and Emel et al. (2021). For wind-pollinated and wind-dispersed *E. vaginatum*, resistance due to forest land cover in the Arctic landscape is expected to be a primary physical factor limiting gene flow. As the extent to which land cover types affect gene flow is unknown, we tested a series of resistance surfaces for Forest, Woodland, and Shrubland categories, assigning combinations of low resistance (2 or 5), medium resistance (10, 20, or 50), and high resistance (100 or 500). Landscape classes in the Other category were assigned a resistance of 1, or lack of resistance. An ENM model based on habitat suitability using MaxEnt output was used to examine IBE. To create a null model, all cells were assigned a resistance cost value of 1, equivalent to an IBD scenario. Pairwise landscape resistance matrices were calculated between sites for 11 different models.

To determine which model best reflected gene flow across sites, we used maximum likelihood of population effects (MLPE) models to test the IBD, IBE, or IBR models with pairwise *F*_*ST*_ genetic distances as response variables and pairwise resistance matrices as explanatory variables. MLPE models implement a type of linear regression on distance matrices while accounting for random effects of pairwise data (Clarke et al., 2002). MLPE models were utilized with modified lmer models in the R package *lme4* v.1.1.27.1 (Bates et al., 2015). Best supported models were identified using AICc. The r.squaredGLMM function in the *mumin* R package v.1.43.17 (Barton, 2018) was used to calculate marginal *R*^2^ values and provide a measure of goodness-of-fit.

### Genotype-Environment Association

As geographic and landscape variables often influence patterns of genomic variation and spatial distribution of plants (Manel et al., 2003; Sork et al., 2013; Lind et al., 2017), genotype-environment association (GEA) methods were implemented to investigate these associations using the comprehensive data set with missing data imputed based on averaged allele frequencies for each site. Nineteen environmental predictors that are important for ecological models in the Arctic (Pearson et al., 2013) were obtained from the WorldClim 2.0 Bioclimatic database (Fick and Hijmans, 2017). We used averages from 1970 to 2000 for GPS locations of sampling sites to distinguish climatic attributes of populations. Global multi-resolution terrain elevation data (GTMED2010; Danielson and Gesch, 2011) was obtained from Google Earth Engine (Gorelick et al., 2017) based on GPS coordinates. In addition, Moran’s eigenvector maps (MEMs) were calculated based on linear distances among sites with the R *adespatial* v.0.0–7 (Dray et al., 2016) and *spdep* v.0.6–9 (Bivand et al., 2013) packages. MEMs were included as predictor variables if their Moran’s I value was associated with significant (100 permutations, *p* < 0.05) allele frequency variance.

Due to high correlation (*R*^2^ > 0.7) of predictor variables (see section “Results”), a principal components analysis (PCA) was conducted with the *prcomp* function of the R *stats* package v.3.6.0 (R Core Team, 2013) to estimate the influence of predictors on principal component (PC) axes and to reduce variables. Two environmental PCAs were run that included temperature and precipitation, which were grouped into two subsets of 11 temperature variables and 8 precipitation variables (Supplementary Table 2). The first two axes were retained for both PCAs, following the Kaiser-Guttman criterion (Guttman, 1954). If several predictor variables strongly influence PC1 and PC2 axes in both PCAs, it can make interpretations of results difficult if environmental variables are summarized (Rellstab et al., 2015), an approach often taken in PCA (Brauer et al., 2018; Wellband et al., 2019). Predictor variables with *R*^2^ > 0.75 and < −0.75 (Schweizer et al., 2016; Forester et al., 2018) and variance inflation factors (VIFs) > 20 were removed (Ter Braak, 1988; Pienitz et al., 1995).

The R *vegan* v.2.5.6 (Oksanen et al., 2013) and *psych* v.1.8.12 (Revelle, 2014) packages were used to run a redundancy analysis (RDA) to investigate co-variation of alleles in response to environmental predictors. Although candidate outlier loci analyses using ddRAD are incomplete as they only sample a fraction of the genome (Lowry et al., 2017), they can be informative for identifying some genes that may have a role in adaptation. The relatively small genome of *E. vaginatum* (1C ≈ 0.4 pg; Rewers et al., 2012) also provides for a higher probability of capturing candidate outlier loci. Site-based allele frequencies used for RDA were computed with *adegenet* v.2.0.1. The RDA was then run using the reduced predictor variable data set (*n* = 7; see section “Results”). Significance of the final models and constrained axes were identified with 999 permutations and a *p*-value of 0.05 (Forester et al., 2018). SNPs that loaded ± 2 *SD* from the mean loading of significant RDA axes were recognized as candidate outlier loci to retain as many potential candidate loci as possible and to discern between neutral loci that share similar spatial signatures to outliers (Forester et al., 2018). The strongest correlations between candidate SNPs and variables were then identified based on the highest correlation coefficients (Forester et al., 2018). Candidate loci found with RDA were investigated using the *blastn* function in BLAST (Altschul et al., 1990), and queried against the NCBI non-redundant nucleotide database and the transcriptome of *E. vaginatum* (Mohl et al., 2020). Subsequently, candidate loci related to local adaptation were annotated with the transcriptome to find the associated Gene Ontology term for each gene based on a percentage identity match of at least 80.0 and an *E*-value threshold of at least 1 × 10^–4^.

## Results

### Genomic Sequence Data and Genomic Diversity

After removal of low-quality reads and reads without radtags, a total of 546,642,395 single-end sequences were retained for 273 individuals, with an average of 2,002,353 sequencing reads per individual. All samples had a coverage depth > 13×. The comprehensive SNP data set contained 3,879 loci and 10,734 SNPs. Twenty-one outlier loci, each with a single SNP, were identified using BayeScan, and these were removed to create a putatively neutral dataset. The neutral data set was comprised of 2,776 loci, each represented by a single SNP, after filtering out SNPs deviating from HWE (*p* ≤ 0.001).

For the neutral SNP data set, global population estimates revealed a mean *F*_*IS*_ = −0.003 (SE = ± 0.001). H_*o*_ (mean = 0.173; SE = ± 0.0008) and H_*e*_ (mean = 0.173; SE = ± 0.0008) varied little among sites (Table 2). Allelic richness (Ar) ranged from 1.590 to 1.667 among sites, with one private allele (in EC) identified (Table 2). *Ne* varied from 39.9 to ∞ across sites, but was generally high except near treeline and for isolated sites in the south.

**TABLE 2:** Genetic diversity summary and demographic statistics for the neutral data set of the 17 *Eriophorum vaginatum* sites sampled in north central Alaska.

Site	*N*	*A*_*r*_	*P*_*a*_	*H*_*o*_	*H*_*e*_	*F*_*IS*_	*Ne*
EC	16	1.590	1	0.171	0.167	–0.025	5923.3
NC	15	1.634	0	0.174	0.173	0.002	202.0
VM	15	1.627	0	0.171	0.171	0.004	39.9
CC	16	1.645	0	0.175	0.174	–0.003	60.9
EL	16	1.658	0	0.178	0.176	–0.014	∞
NN	16	1.661	0	0.177	0.178	0.004	∞
GO	16	1.663	0	0.173	0.177	0.023	∞
CF	18	1.667	0	0.184	0.177	–0.036	∞
ST	16	1.645	0	0.170	0.175	0.029	186.4
TB	16	1.632	0	0.166	0.171	0.028	221.4
CH	16	1.643	0	0.179	0.175	–0.029	314.5
AT	16	1.634	0	0.171	0.172	0.011	∞
TL	16	1.627	0	0.171	0.169	–0.011	∞
AN	16	1.627	0	0.167	0.169	0.017	∞
SG	17	1.645	0	0.174	0.173	–0.006	∞
CP	16	1.656	0	0.172	0.175	0.019	∞
PB	16	1.653	0	0.173	0.175	0.013	∞

*Sites are ordered geographically from South to North and site abbreviations follow Table 1. Sample size (N), allelic richness (A_r_), private alleles (P_a_), observed heterozygosity (H_o_), expected heterozygosity (H_e_), inbreeding coefficient (F_IS_) and effective population size (Ne).*

### Population Structure

On average, we attained a global *F*_*ST*_ of 0.020, with pairwise values generally being lower among sites above treeline (0.002–0.010; mean = 0.006) than below treeline (excluding EC; 0.004–0.026; mean = 0.014). Eagle Creek had relatively high *F*_*ST*_ values in all pairwise comparisons (0.034–0.060; mean = 0.047). While all pairwise comparisons were significant (*p* < 0.01) between sites below treeline, this was not true between some sites above treeline (Supplementary Table 3). AMOVA results demonstrated that a high percentage of neutral genetic variation occurred within sites (94.9%), however, variation was significant among all comparisons (Supplementary Table 4).

**FIGURE 3:**
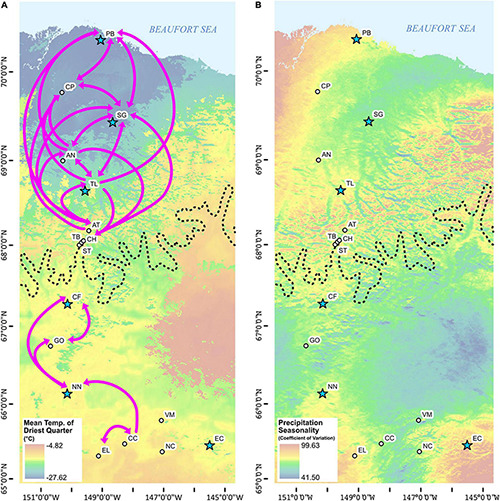
Maps of the study area with **(A)** mean temperature of the driest quarter (tdq) and **(B)** precipitation seasonality (prs) WorldClim underlying data layers using ARCMAP v.10.7.1 (ESRI, 2011). Note that *divMigrate* migration values ≥ 0.80 and direction indicated with arrows are also provided in **(A)**. Site abbreviations as for Table 1.

Highest *divMigrate G*_*ST*_ migration values (≥ 0.80) were found between sites north of treeline (CH, AT, TL, AN, SG, CP, PB) and between CC, EL, NN, GO, and CF south of treeline (Figure 3 and Supplementary Table 5), with a break at treeline. Migration values were lower for sites sampled adjacent to the treeline boundary and among more isolated higher elevation sites (EC, NC, and VM) south of treeline. Migration values from SG into other northern sites were consistently higher than from other sites (Figure 3 and Supplementary Table 5). Asymmetric gene flow was found between most sites, but values were not significant.

**FIGURE 4:**
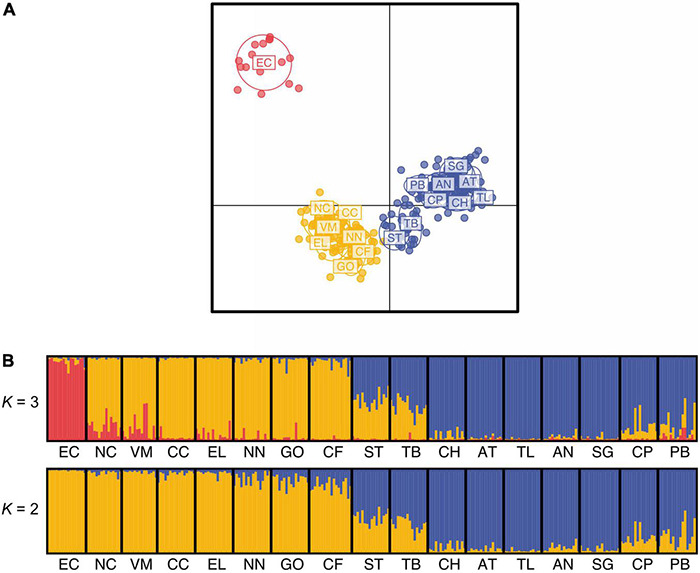
Population structure results from the neutral SNP data set. **(A)** Scatter plot from the discriminant analysis of principal components (DAPC) with 80 principal components retained on the first two Discriminant Analysis axes showing the differentiation between the three groups and inertia ellipses. Each color represents a cluster as identified with the Bayesian Information Criterion (BIC). Red dots represent individuals from the Eagle Creek (EC) population, yellow dots represent individuals from the population south of treeline except for EC (South), and blue dots represent individuals from populations north of treeline (North). **(B)** Bar graph of STRUCTURE results for population structure analysis. Each vertical bar represents an individual, and colors show the proportion of ancestry assigned to each of the three clusters (*K* = 2 and *K* = 3), as inferred from Δ*K* values. *Eriophorum vaginatum* populations are ordered from south to north location along the latitudinal gradient in north central Alaska. See Table 1 for collection site abbreviations.

The best Δ*K* value resulting from STRUCTURE analyses was *K* = 2 (Supplementary Figure 1A). However, the BIC score for *K* = 3 from DAPC analyses was optimal (Supplementary Figure 1B). Populations sampled north or south of treeline formed clusters at both *K* = 2 or *K* = 3 for DAPC and STRUCTURE analyses (Figure 4). At *K* = 3, EC was identified as a unique cluster in the DAPC scatter plot and in barplots of STRUCTURE results. Δ*K* methods, in particular, have been shown to bias toward *K* = 2 for populations with subtle population structure and in simulated data when *K* = 1 or *K* = 3 were supported for simulations (Cullingham et al., 2020). In this case, EC was also differentiated by high *F*_*ST*_ values in pairwise comparisons and low *G*_*ST*_ based migration values compared to other populations. Populations on either side of treeline (ST and TB) showed evidence of admixture based on both STRUCTURE and DAPC results (Figure 4).

### Demographic and Environmental Niche Modeling

**FIGURE 5:**
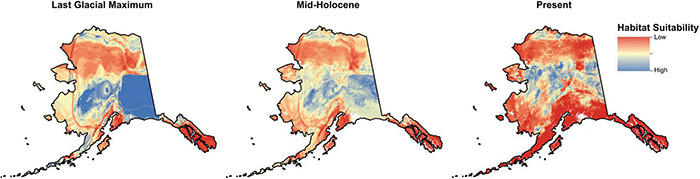
MaxEnt environmental niche model (ENM) maps of Alaska, United States adapted to depict *Eriophorum vaginatum* habitat suitability during the Last Glacial Maximum (LGM), Mid-Holocene and present. Modeled LGM layers were derived from the Community Climate System Model (CCSM) (Braconnot et al., 2007). Current and LGM layer habitat suitability bin values were averaged to create the Mid-Holocene climate raster.

The contemporary species distribution model and climate scenario projections from the LGM (∼20 kyr BP) identified suitable habitat throughout the Beringian refugium for *E. vaginatum* (Figure 5) outside of the glaciated regions within the refugium (notably including north and south of the Brooks Range). Projection from the mid-Holocene (∼6 kyr BP) shows suitable habitat extended throughout most of northern Alaska and was continuous across the formerly glaciated Brooks Range. MLPE models incorporating increased resistance for forest, woodland, and shrubland supported IBR as an important factor in shaping neutral genetic structure across sites. The best MLPE models favored higher forest resistance compared to other categories, and higher resistance from woodland than shrubland was also important. The best IBR models explained ∼21% of the genetic variation, which was ∼17% more than the variation explained by the null IBD model (Table 3). Including IBE (ENM) did not improve the best IBR models, but IBE alone and models incorporating IBE also outperformed the IBD model in all measures.

**TABLE 3:** Maximum likelihood of population effects (MLPE) models relating pairwise *F*_*ST*_ to pairwise distance matrices of isolation by resistance (IBR), isolation by environment (IBE), and isolation by distance (IBD) and ranked by AICc.

Resistance values						
**Model**	**Forest**	**Woodland**	**Shrubland**	**AICc**	**ΔAICc**	***R*^2^m**
IBR	100	50	20	–1045.7	0	0.21
IBR	500	50	20	–1029.1	16.6	0.21
IBR	10	5	2	–1024.5	21.2	0.21
IBR	100	50	50	–1021.2	24.5	0.17
IBR	100	100	20	–1012.5	33.2	0.18
IBR + IBE	100	50	20	–1002.1	43.6	0.15
IBR	100	50	2	–997.1	48.6	0.17
IBR + IBE	500	50	20	–995.9	49.8	0.18
IBR + IBE	10	5	2	–991.6	54.1	0.14
IBE	–	–	–	–991.2	54.5	0.07
IBD	–	–	–	–965.1	80.6	0.04

*R^2^m = marginal R^2^ approximation of mixed model fixed effects.*

### Genotype-Environment Association

Two environmental PCAs were run: (1) temperature and (2) precipitation. PC1 and PC2 explained 90.5% of the total variation of the temperature PCA and 96.0% of the precipitation PCA. All predictors strongly influenced both PC1 and PC2, so environmental predictors were not grouped into summarized environmental variables. The retained variables explaining significant allelic variation for the RDA analysis were: (1) isothermality (iso; annual mean diurnal range/annual temperature range), (2) mean temperature of the driest quarter (tdq; Feb–Apr), (3) mean temperature of the warmest quarter (twq; Jun–Aug), (4) precipitation of the driest month (pdm; Apr), (5) precipitation seasonality (prs; coefficient of variation estimated from the standard deviation of monthly precipitation estimates), (6) MEM1, and (7) MEM2 (Supplementary Table 6).

The RDA model was significant (*p* = 0.001), and predictor variables explained 13.2% of the total genetic variance. The RDA1 discriminant axis was significant (*p* = 0.001), and explained 30.1% of the constrained variation. The RDA2 axis was not significant (*p* = 0.070). There were 165 candidate SNPs identified and detected on the RDA1 axis and 162 candidate SNPs with high correlations (*R*^2^ ≥ 0.7) to predictor variables, which were retained to further investigate local adaptation (Table 4 and Supplementary Table 7). Of the candidate SNPs with correlations *R*^2^ ≥ 0.7 to predictor variables, most were correlated with the MEM1 variable (141 SNPs). The remaining candidate SNPs were correlated with tdq (11 SNPs) and twq (10 SNPs). Gene Ontology annotations were found for 45 candidate loci after a search against the *E. vaginatum* transcriptome (Mohl et al., 2020; Table 4 and Supplementary Table 7).

**TABLE 4:** Annotated *Eriophorum vaginatum* candidate genes with a percentage identity match of at least 80.0 and an *E*-value threshold of at least 1 × 10^–4^ and RDA *R*^2^ value ≥ 0.8.

Locus ID	Gene	Protein	GO functional term	RDA R^2^	RDA Predictor	2′ RDA predictor	*E*-value	Similarity%
42946	EMB8	Embryogenesis-associated EMB8	MF	0.933	MEM1	N/A	1.00E-70	100
68081	HIPP26*	Heavy metal-associated isoprenylated plant protein 26-like	MF, BP	0.911	MEM1	N/A	4.00E-71	99
2666657	VPS37	Vacuolar-sorting-associated 37 homolog 1-like	MF, BP	0.907	MEM1	N/A	4.00E-71	100
27083	LRR*	Probable LRR receptor-like serine/threonine-protein kinase	MF	0.906	tdq	MEM1	4.00E-71	100
53674	TMEM33	Transmembrane 33 homolog	BP	0.898	MEM1	N/A	1.00E-21	91
57279	pKIWI502	fruit protein pKIWI502-like	MF	0.897	MEM1	N/A	2.00E-69	99
61802	CEK2*	Probable choline kinase 2	MF	0.893	MEM1	N/A	4.00E-71	100
47488	ASAT	Putative aspartate aminotransferase	MF, BP	0.891	tdq	MEM1	3.00E-77	99
1469	ADH2*	Alcohol dehydrogenase class-3	MF, BP	0.874	MEM1	N/A	8.00E-68	99
3039887	GRF2	14-3-3-like protein GF14 omega	BP	0.871	MEM1	N/A	1.00E-71	100
63730	At2g45590*	Receptor-like serine threonine-kinase At2g45590	MF	0.865	MEM1	N/A	2.00E-69	99
11498	CBP	Serine carboxypeptidase	MF	0.847	MEM1	N/A	4.00E-71	100
33151	SIZ1*	E3 SUMO-ligase SIZ1 isoform X1	MF	0.846	twq	MEM1	4.00E-71	100
38057	RPC3	DNA-directed RNA polymerase III subunit RPC3	MF, BP	0.845	MEM1	N/A	4.00E-71	100
1151144	POLD2	DNA polymerase delta small subunit	MF, BP	0.841	MEM1	tdq	4.00E-71	100
38483	PISD*	Phosphatidylserine decarboxylase proenzyme 1, mitochondrial-like	MF, BP	0.837	MEM1	N/A	2.00E-69	99
4094	EIL1A*	Ethylene Insensitive 3-like 1	MF, BP	0.829	MEM1	N/A	4.00E-71	100
26505	FC2*	Ferrochelatase-2, chloroplastic	MF, BP	0.827	MEM1	N/A	4.00E-71	100
45291	ABCB*	ABC transporter B family member 13-like isoform	MF	0.823	MEM1	N/A	4.00E-71	100
4623	VIP6	CTR9 homolog	MF, BP	0.823	MEM1	N/A	1.00E-71	100
47238	AHK*	Histidine kinase	MF, BP	0.821	MEM1	N/A	4.00E-71	100
1847	CRR7	Chlororespiratory Reduction 7, chloroplastic	MF, BP	0.811	MEM1	N/A	3.00E-07	97
69795	ZBED1	BED zinc finger, hAT family dimerization domain isoform 1	MF, BP	0.811	MEM1	N/A	3.00E-27	92
45293	UBP27*	Ubiquitin carboxyl-terminal hydrolase 27 isoform	MF, BP	0.810	MEM1	N/A	4.00E-71	100
70778	ACO1	1-aminocyclopropane-1-carboxylate oxidase homolog 1-like	MF	0.808	MEM1	N/A	2.00E-68	98

RDA Predictors had the highest R^2^ values for that association with the given gene. 2′ RDA Predictors had R^2^ values > 0.7 and are listed from highest to lowest. E-value and Similarity% for gene ID of each locus. MF, Molecular Function; and BP, Biological Process. *Genes with stress response association.

## Discussion

### Landscape Genomics of an Arctic Foundation Species

Three genetic clusters were identified for sampled *E. vaginatum* (Figure 4; EC, North, and South) across a latitudinal gradient encompassing two ecosystems in the north-central Alaskan Arctic. Most populations genetically clustered by geographical locations north or south of treeline (Figures 1, 4 and Table 1) with the exception of treeline adjacent populations (ST and TB), which had a near equal genetic assignment to both north or south genetic clusters, and EC, which formed its own genetic cluster. Importantly, population structure results support the presence of glacial barriers to gene flow for *E. vaginatum*, and corroborates long-term studies finding adaptations shared among plants north vs. south of the Brooks Range (Fetcher and Shaver, 1990; Bennington et al., 2012).

Post-glaciation, the Brooks Range could have provided an allopatric barrier for *E. vaginatum* colonization. However, our landscape-level sampling suggests the gene flow barrier is at treeline, well below the summit of the range. The Continental Divide defines a north and south slope through the Brooks Range and populations above treeline from the south slope of the Brooks Range (i.e., CH) clusters with north slope populations while south slope treeline adjacent populations ST (south of treeline) and TB (north of treeline) show evidence of an admixture zone only at treeline. While treeline in the Arctic signifies a contemporary ecosystem change, the arctic flora was also shaped in part by patterns of glaciation and distribution of refugia. The Beringian refugium was an important source for post-glaciation vegetation, but in Alaska it was divided by glaciation over the Brooks Range (Figure 1) that has likely affected the genetic structure of the Alaskan arctic flora. Here, we propose a hypothesis for the contemporary genetic structure of *E. vaginatum* that could have implications for the adaptations found in long-term studies of plants in our transect. Two subrefugia were present north and south of the Brooks Range glaciation (Figure 5), with each population accumulating genetic variation in allopatry via genetic drift and environmental adaptation; this was followed by post-glaciation advancement. Both geophysical attributes and climatic events in this region of Alaska support the idea of two centers of origin for *E. vaginatum*. The Brooks Range remained glaciated throughout the Pleistocene glacial cycles (Figure 1; Kaufman and Manley, 2004; Kaufman et al., 2011), creating a formidable barrier to gene flow between northern and southern regions of the eastern Beringian refugium. Pollen of *E. vaginatum* has been found in Yedoma sediment samples, indicating its presence in both regions during paleoclimate fluctuations encompassing the LGM (Schirrmeister et al., 2016; Lapointe et al., 2017). Likewise, pollen profiles in central and northern Alaska during the last ∼36 kyr suggest a flora in the region similar to that found today, with dominance of graminoids in the north and spruce woodlands in the south (Billings, 1992; Finkenbinder et al., 2014; Schirrmeister et al., 2016; Lapointe et al., 2017). Demographic analyses suggest that at the glacial maximum environmental conditions in the two regions were suitable for *E. vaginatum* as well (Figure 5). As such, *E. vaginatum* (along with the rest of the flora) had the opportunity to adapt to contrasting abiotic and biotic factors reflecting the contemporary ecosystems that now have a transition zone at the treeline ecotone.

**FIGURE 6:**
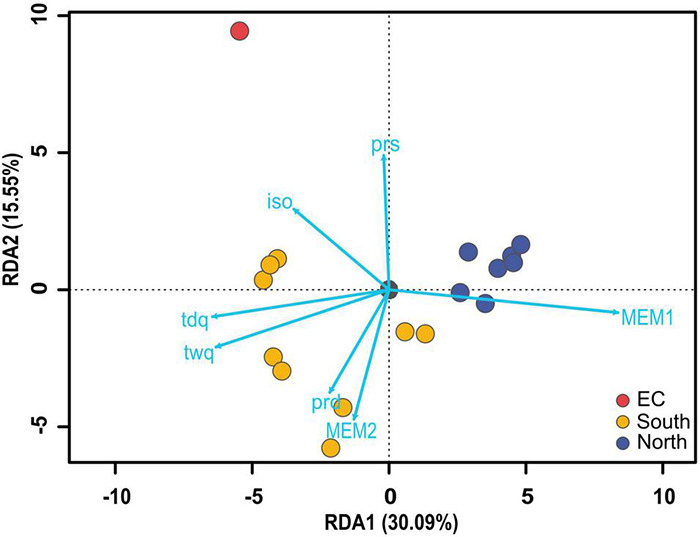
RDA plot demonstrating predictor associations as related to ecotype for the comprehensive SNP data set. iso, Isothermality; tdq, Mean temperature of the driest quarter (Feb, Mar, Apr), twq, Mean temperature of the warmest quarter (Jun, Jul, Aug), prd, Precipitation of the driest month (Apr), prs, Precipitation seasonality.

Adaptive variation is supported by GEA analyses that indicate environmental predictors (Figure 6) contribute to allelic turnover with a shift related to changes at treeline (Supplementary Table 6), corroborating evidence from long-term ecological studies (e.g., Fetcher and Shaver, 1990; Bennington et al., 2012). Genetic structure would also be reinforced through neutral variation accumulated during the period of allopatry caused by glacial isolation surrounding the LGM. IBR based on vegetation cover, especially denser forest, also has a large effect on contemporary gene flow and probably has a large effect on the genetic structure we found here. A particularly important consequence of IBR due to forest density is its role in preventing future gene flow north, as potentially better adapted ecotypes from the south will have restrained capacity to migrate north. In summary, both geographic isolation and adaptation influenced the genetic structure that is now delimited at treeline, as supported by demographic modeling analyses, which identified that both IBR and IBE had a more significant role in restricting gene flow than IBD (Table 3).

Migration patterns also support bidirectional gene flow from the proposed subrefugia. The *divMigrate* analyses indicate gene flow to the southernmost site above treeline (i.e., CH) on the south slope of the Brooks Range coming from SG, which is located just beyond the northern extent of the LGM (Figures 1, 3). Between June and July, when *E. vaginatum* flowers and fruits, prevailing daytime winds are from the north in this region (Zhang et al., 2016; Environmental Data Center Team, 2022) providing a mechanism for gene flow over the Continental Divide. SG also is the northern population with highest gene flow to all other northern sites, indicating its likely importance as a source population post-glaciation. It is also clear that connectivity and gene flow is high throughout the north slope and coastal plain (Supplementary Table 3), probably due to the lack of the primary IBR variable of forest cover. These results suggest that this region could to some extent be treated as a large population with high genetic connectivity. The highest migration values among South populations are from sites just south of treeline, including GO, the northernmost site beyond the southern glacial boundary at the LGM. Thus, potential source populations on either side of the Brooks Range glaciation likely had a role in colonizing to treeline where gene flow was restricted. Further, logistically challenging sampling along a longitudinal transect above treeline and along the south slope of the Brooks Range would further verify the geographic extent of these results. The denser forest cover and more disjunct *E. vaginatum* population along the southern latitudinal gradient would lead to the lower genetic connectivity of these populations. At the treeline ecotone migration values were low and the two sites sampled at the treeline boundary (i.e., ST and TB) had similar migration values from North and South clusters (Supplementary Table 5). If gene flow proceeded from subrefugia rather than an ancestral gene pool, then the admixed samples at treeline identified in population structure analyses (Figure 4) signify an admixture zone along the treeline ecotone, which further supports treeline as a gene flow barrier. Our modeling of IBR suggests that land-cover resistance continues to have a strong effect on gene flow.

Alternatively, the environmental shift accompanying the moving treeline could have led to rapid evolution during post-glacial expansion if seed banks from widespread species like *E. vaginatum*, that innately carry high allelic diversity, are already present in regions with retreating glaciers (Alsos et al., 2007); and thus, provide the standing genetic variation for rapid adaptive change (Hermisson and Pennings, 2005; Dlugosch et al., 2015). Although such a scenario has been hypothesized for *E. vaginatum* in alpine environments (Walker et al., 2019), the known geological and vegetation history of the region, along with *divMigrate* gene flow patterns (Figure 3) and evidence of population structure (Figure 4) identified here makes this alternative unlikely.

Much of the Arctic has been revegetated relatively recently from refugia, which have typically been represented as a genetic pool without considering adaptive variation within each refugium (Tremblay and Schoen, 1999; Abbott et al., 2000; Alsos et al., 2005, 2007). If *E. vaginatum* from northern and southern Beringian subrefugia represent adaptive ecotypes, as suggested by the long-term ecological studies (Bennington et al., 2012) and molecular data (Mohl et al., 2020; and these results), that colonized formerly glaciated regions, then we would expect them to have more likely colonized ecosystems to which they are best adapted. For example, *E. vaginatum* populations from north of treeline have lower phenotypic plasticity (Fetcher and Shaver, 1990; Parker et al., 2017) and are less responsive in GPP (Curasi et al., 2019) compared to those south of treeline, which could affect persistence of each ecotype as the Arctic warms. Similarly, we provide evidence of allelic turnover in GEA analyses for populations of *E. vaginatum*, suggesting that populations in these two regions are not simply genetically differentiated due to genetic drift in isolation, but have adapted to unique genetic niche space. Together, the data demonstrates the importance of determining the extent and cause for genetic divergence between populations, which is particularly critical when developing species distribution models for climate change (Massatti and Knowles, 2016; Ortego and Knowles, 2020) that include foundation species such as *E. vaginatum*. Attempts to verify the distribution of ecotypes and the adaptive potential of *E. vaginatum* will require expanding landscape genomic studies across entire Arctic ecosystems including refugial regions, and building on the ground-breaking genomic biodiversity work of Wang et al. (2021).

Finally, the site EC, occurring in the southern extent of the sampling range, was identified as genetically distinct (Figure 4), which was unexpected given the similarity in ecological attributes of EC and other populations south of treeline (Fetcher and Shaver, 1990; Bennington et al., 2012). Due to higher landscape resistance south of treeline, populations of *E. vaginatum* are more sparsely distributed with lower genetic connectivity (Figure 3 and Supplementary Table 5). The EC site is at the southern margin of the modeled LGM extent of the Beringian refugium (Figure 1; Kaufman and Manley, 2004; Kaufman et al., 2011). Given the population disjunction and potential for isolation due to glaciation patterns, genetic differentiation of EC could be due to bottlenecking or a founder event as supported by this population having the lowest allelic richness and low observed heterozygosity, as compared to the other sampled sites (Table 2). However, effective population size for EC was relatively high (*Ne* = 5923.3) and it is not uniquely isolated among southern populations. Thus, we alternatively posit that EC may represent a population adapted to alternative niche space as supported by GEA analyses that uncovered association between allelic turnover and precipitation variables (iso and prs) for this population relative to others (Figures 3B, 6 and Table 4). While our GEA analyses didn’t uncover specific genes correlated to iso and prs, Mohl et al. (2020) found that EC had differential expression of abiotic stress genes not found in other populations along the latitudinal distribution. Future work will benefit from examining southern populations with similar precipitation patterns to further elucidate the importance of adaptive variation for this and other similar populations that may have been uniquely isolated during glacial periods.

### Environmental Heterogeneity Explains Allelic Turnover Across a Latitudinal Cline

The Arctic covers roughly 7% of the Earth’s surface area or 14% of its land area, and its vegetation history has been shaped by recurring patterns of glaciation, geological landscape structure, and environmental shifts in attributes such as permafrost depth (Billings, 1973; Meyerhoff and Meyerhoff, 1973). Likewise, the transitions associated with treeline in the Arctic aren’t necessarily abrupt; instead there is a cline that could require adaptations that can range from local specificity to wide-ranging plasticity (Fetcher and Shaver, 1990; Bennington et al., 2012; Curasi et al., 2019). This has been exemplified in long-term reciprocal transplant studies that have shown adaptive differences in *E. vaginatum* are complex and related not only to position north or south of treeline, but also to home site and latitude (Shaver et al., 1986; Fetcher and Shaver, 1990; Bennington et al., 2012; Curasi et al., 2019; Mohl et al., 2020). As expected, a genetic signature was recovered related to environment along the cline of the latitudinal gradient, not just at treeline. Evidence for environmental association with allele frequency turnover was found for all predictors in the RDA: MEM1, temperature, and precipitation (Figure 6). RDA1 was correlated with most environmental variables considered and corresponded with the distribution of populations along the latitudinal gradient.

The primary temperature predictors (tdq and twq) relate to spring and summer conditions, which encompass the limited growing season of *E. vaginatum* in the Arctic and show a clinal change in both mean and variance along the latitudinal gradient (Supplementary Table 1). The short growing season for most plants in the Arctic begins in late spring following snowmelt, as air temperatures and photosynthetic rates increase. Photosynthetic rate then remains relatively high from mid-June to mid-August (Defoliart et al., 1988). Consequently, late spring and summer temperature differences are likely critical for vegetative phenology of *E. vaginatum* (Siegenthaler et al., 2013; Parker et al., 2017, 2021). Similar physiological adaptations have been observed for other arctic plant species, especially graminoids, forbs, and deciduous shrubs (Chapin, 1987; Chapin and Shaver, 1996); thus, selective pressures could lead to similar adaptation for co-occurring widespread arctic species.

Several potentially adaptive SNPs (162 candidate SNPs and 131 loci with high correlations to predictor variables; *R*^2^ ≥ 0.7) were identified with GEA methods. MEM1, tdq, and twq, in succession, were the predictors for the 45 loci identified to gene and function (Table 4). MEM variables can represent unmeasured environmental predictors, have a spatial component (Manel et al., 2010; Fitzpatrick and Keller, 2015), and provide important predictive value for allele frequency turnover (Martins et al., 2018; Gibson and Moyle, 2020) included among other arctic-alpine plants (Manel et al., 2012; Bothwell et al., 2013). These three variables most frequently correlated with candidate loci that have roles in abiotic stress response (Table 4). These include transcription factors belonging to gene families established in stress response pathways (e.g., LRR, SIZ1, and EIL; Yeh et al., 2012; Liu et al., 2020; Poór et al., 2021). The importance of these genes is predictable due to the need of plants to adjust to extreme fluctuations in temperature in the low Arctic throughout the growing season (see Supplementary Table 1) and should be the starting point in investigating genes attributing to ecotype adaptation.

In summary, genomic structure and differentiation were identified across 17 *E. vaginatum* sites along a ∼426 km latitudinal gradient in north central Alaska, which was within the boundary of the Beringian refugium. A major genetic boundary was found at the treeline ecotone. Strong evidence of IBR was found across the study region, highlighting the influence of vegetation type and density on *E. vaginatum* neutral genetic structure. Importantly, IBR due to forest cover can signify a major obstacle for gene flow of potentially better-adapted genotypes northward with rapid climate change. GEA results support ecotypic adaptations elucidated over decades of ecological studies for this arctic foundation species and suggest potential important environmental variables along with candidate loci, many of which are associated to abiotic stress gene pathways. These results are consequential for increasing predictive accuracy of distribution changes under climate change. An understanding of adaptive variation should be incorporated into developing hybrid environmental niche and species distribution models (Ikeda et al., 2017a; Razgour et al., 2019; Waldvogel et al., 2020). The combination of broader sampling and genomic studies of other foundational arctic plants will increase understanding of local adaptation, gene flow, and environmental associations crucial for determining the long-term consequences of climate change in the arctic flora. We conclude that accounting for ecotypic physiology, gene flow, local adaptation and gene expression of foundation species under changing climates will lead to a greater understanding of response from the level of the individual to the ecosystem.

## Data Availability Statement

The datasets presented in this study can be found in online repositories. The names of the repository/repositories and accession numbers can be found below: https://www.ncbi.nlm.nih.gov/, BioProject PRJNA803172, accession numbers SAMN25639742–SAMN25640014. The STACKS pipeline Python script, Redundancy Analysis R code, and ENMEVAL R code can be found at https://github.com/estunz/EvLG2022.

## Author Contributions

MM, NF, and JT designed the study. MM, ES, and NF organized and performed field collections. ES and MM wrote the manuscript. ES performed lab research and analyzed the data. JM contributed bioinformatic analytical tools and training and assisted with data analyses. PL provided ddRAD data collection techniques, lab resources, and assistance. All authors reviewed and approved the final manuscript.

## Conflict of Interest

The authors declare that the research was conducted in the absence of any commercial or financial relationships that could be construed as a potential conflict of interest.

## Publisher’s Note

All claims expressed in this article are solely those of the authors and do not necessarily represent those of their affiliated organizations, or those of the publisher, the editors and the reviewers. Any product that may be evaluated in this article, or claim that may be made by its manufacturer, is not guaranteed or endorsed by the publisher.

## Footnotes

1
https://zzz.bwh.harvard.edu/plink/download.shtml
