# Supplementary material for: Landscape Genomics Provides Evidence of Ecotypic Adaptation and a Barrier to Gene Flow at Treeline for the Arctic Foundation Species Eriophorum vaginatum
Source: Front Plant Sci. 2022 Mar 24;13:860439. doi: 10.3389/fpls.2022.860439 (PMC8987161; doi:10.3389/fpls.2022.860439)
Supplement: Supplementary file 1 [file Table_1.DOCX]

**Supplementary Table S1.** Mean annual temperature and mean annual rainfall (obtained from the WorldClim 2.0 Bioclimatic database (Fick and Hijmans, 2017)). 17 site abbreviations as for **Table 1**.

| **Site** | **Minimum-Maximum Mean Temperature Range (°C)** | **Mean Annual Temperature (°C)** | **Mean Annual Precipitation (mm)** |
| --- | --- | --- | --- |
| **Eagle Creek (EC)** | -28.30° - 18.40° | -4.94° | 247 |
| **Nome Creek (NC)** | -29.90° - 20.10° | -4.87° | 319 |
| **Victoria Mountain (VM)** | -25.20° - 17.30° | -4.55° | 290 |
| **Colorado Creek (CC)** | -30.30° - 20.90° | -4.09° | 288 |
| **Elliott Highway (EL)** | -25.30° - 17.80° | -3.98° | 311 |
| **No Name Creek (NN)** | -31.20° - 21.90° | -5.07° | 334 |
| **Gobbler’s Knob (GO)** | -27.70° - 18.70° | -4.98° | 348 |
| **Coldfoot (CF)** | -31.20° - 19.80° | -5.58° | 297 |
| **South of Timberline (ST)** | -27.70° - 15.10° | -6.97° | 279 |
| **Timberline (TB)** | -28.00° - 15.60° | -6.92° | 282 |
| **Chandalar (CH)** | -26.90° - 14.10° | -7.55° | 288 |
| **Atigun Camp (AT)** | -25.40° - 11.50° | -8.75° | 279 |
| **Toolik Lake (TL)** | -29.30° - 16.00° | -8.74° | 239 |
| **Anaktuvuk (AN)** | -32.90° - 18.10° | -9.72° | 210 |
| **Sagwon (SG)** | -34.00° - 17.70° | -10.08° | 183 |
| **Coastal Plain (CP)** | -33.10° - 15.80° | -10.37° | 171 |
| **Prudhoe Bay (PB)** | -31.60° - 11.80° | -11.35° | 161 |
