# Supplementary material for: Landscape Genomics Provides Evidence of Ecotypic Adaptation and a Barrier to Gene Flow at Treeline for the Arctic Foundation Species Eriophorum vaginatum
Source: Front Plant Sci. 2022 Mar 24;13:860439. doi: 10.3389/fpls.2022.860439 (PMC8987161; doi:10.3389/fpls.2022.860439)
Supplement: Supplementary file 2 [file Table_2.DOCX]

**Supplementary Table S2.** Variable subsets of temperature and precipitation variables (from the WorldClim 2.0 Bioclimatic database (Fick and Hijmans, 2017) used in Principal Components Analysis (PCA). Mean diurnal range = mean of monthly maximum temperature-minimum temperature, Isothermality = annual mean diurnal range/annual temperature range, Temperature seasonality (standard deviation * 100), Maximum temperature of the warmest month (Jul), Minimum temperature of the coldest month (Jan), Temperature annual range (maximum temperature of the warmest month (Jul)-minimum temperature of the coldest month (Jan), Mean temperature of the wettest quarter (Jul, Aug, Sept), Mean temperature of the driest quarter (Feb, Mar, Apr), Mean temperature of the warmest quarter (Jun, Jul, Aug), Mean temperature of the coldest quarter (Dec, Jan, Feb), Precipitation of the wettest month (Aug), Precipitation of the driest month (Apr), Precipitation seasonality = coefficient of variation estimated from the standard deviation of monthly precipitation estimates; Precipitation of the wettest quarter (Jul, Aug, Sept), Precipitation of the driest quarter (Feb, Mar, Apr), Precipitation of the warmest quarter (Jun, Jul, Aug), Precipitation of the coldest quarter (Dec, Jan, Feb).

| **Temperature Variables** | **Precipitation Variables** |
| --- | --- |
| Annual mean temperature (°C) | Annual precipitation (mm) |
| Mean diurnal range (°C) | Precipitation of the wettest month (mm) |
| Isothermality (%) | Precipitation of the driest month (mm) |
| Temperature seasonality (°C) | Precipitation seasonality (coefficient of variation) |
| Maximum temperature of the warmest month (°C) | Precipitation of the wettest quarter (mm) |
| Minimum temperature of the coldest month (°C) | Precipitation of the driest quarter (mm) |
| Temperature annual range (°C) | Precipitation of the warmest quarter (mm) |
| Mean temperature of the wettest quarter (°C) | Precipitation of the coldest quarter (mm) |
| Mean temperature of the driest quarter (°C) |  |
| Mean temperature of the warmest quarter (°C) |  |
| Mean temperature of the coldest quarter (°C) |  |
