# Supplementary material for: Landscape Genomics Provides Evidence of Ecotypic Adaptation and a Barrier to Gene Flow at Treeline for the Arctic Foundation Species Eriophorum vaginatum
Source: Front Plant Sci. 2022 Mar 24;13:860439. doi: 10.3389/fpls.2022.860439 (PMC8987161; doi:10.3389/fpls.2022.860439)
Supplement: Supplementary file 3 [file Table_3.DOCX]

**Supplementary** **Table S3.** Table of Pairwise *F*_ST_ values for the neutral dataset. *F*_ST_ values are on the lower diagonal and p-values are on the upper diagonal. * refers to significance at ≤ 0.01.

|  | EC | NC | VM | CC | EL | NN | GO | CF | ST | TB | CH | AT | TL | AN | SG | CP | PB |
| --- | --- | --- | --- | --- | --- | --- | --- | --- | --- | --- | --- | --- | --- | --- | --- | --- | --- |
| EC |  | 0.001* | 0.001* | 0.001* | 0.001* | 0.001* | 0.001* | 0.001* | 0.001* | 0.001* | 0.001* | 0.001* | 0.001* | 0.001* | 0.001* | 0.001* | 0.001* |
| NC | 0.034 |  | 0.001* | 0.002* | 0.001* | 0.001* | 0.001* | 0.001* | 0.001* | 0.001* | 0.001* | 0.001* | 0.001* | 0.001* | 0.001* | 0.001* | 0.001* |
| VM | 0.039 | 0.012 |  | 0.001* | 0.001* | 0.001* | 0.001* | 0.001* | 0.001* | 0.001* | 0.001* | 0.001* | 0.001* | 0.001* | 0.001* | 0.001* | 0.001* |
| CC | 0.039 | 0.008 | 0.015 |  | 0.001* | 0.001* | 0.001* | 0.001* | 0.001* | 0.001* | 0.001* | 0.001* | 0.001* | 0.001* | 0.001* | 0.001* | 0.001* |
| EL | 0.039 | 0.008 | 0.013 | 0.008 |  | 0.001* | 0.001* | 0.001* | 0.001* | 0.001* | 0.001* | 0.001* | 0.001* | 0.001* | 0.001* | 0.001* | 0.001* |
| NN | 0.038 | 0.009 | 0.012 | 0.008 | 0.010 |  | 0.001* | 0.001* | 0.001* | 0.001* | 0.001* | 0.001* | 0.001* | 0.001* | 0.001* | 0.001* | 0.001* |
| GO | 0.041 | 0.012 | 0.015 | 0.011 | 0.011 | 0.005 |  | 0.001* | 0.001* | 0.001* | 0.001* | 0.001* | 0.001* | 0.001* | 0.001* | 0.001* | 0.001* |
| CF | 0.043 | 0.011 | 0.017 | 0.014 | 0.012 | 0.006 | 0.004 |  | 0.001* | 0.001* | 0.001* | 0.001* | 0.001* | 0.001* | 0.001* | 0.001* | 0.001* |
| ST | 0.050 | 0.021 | 0.025 | 0.023 | 0.021 | 0.016 | 0.015 | 0.015 |  | 0.001* | 0.001* | 0.001* | 0.001* | 0.001* | 0.001* | 0.001* | 0.001* |
| TB | 0.054 | 0.022 | 0.026 | 0.022 | 0.023 | 0.018 | 0.014 | 0.016 | 0.017 |  | 0.001* | 0.001* | 0.001* | 0.001* | 0.001* | 0.001* | 0.001* |
| CH | 0.054 | 0.025 | 0.029 | 0.025 | 0.025 | 0.021 | 0.022 | 0.019 | 0.018 | 0.016 |  | 0.001* | 0.001* | 0.001* | 0.001* | 0.001* | 0.001* |
| AT | 0.054 | 0.026 | 0.031 | 0.028 | 0.024 | 0.023 | 0.023 | 0.019 | 0.019 | 0.015 | 0.008 |  | 0.001* | 0.003* | 0.020 | 0.001* | 0.001* |
| TL | 0.060 | 0.027 | 0.033 | 0.029 | 0.027 | 0.024 | 0.026 | 0.024 | 0.018 | 0.019 | 0.008 | 0.006 |  | 0.004* | 0.078 | 0.001* | 0.001* |
| AN | 0.054 | 0.021 | 0.027 | 0.025 | 0.022 | 0.021 | 0.021 | 0.019 | 0.015 | 0.014 | 0.006 | 0.004 | 0.004 |  | 0.003* | 0.001* | 0.001* |
| SG | 0.052 | 0.025 | 0.029 | 0.023 | 0.027 | 0.021 | 0.024 | 0.022 | 0.017 | 0.016 | 0.008 | 0.003 | 0.002 | 0.004 |  | 0.030 | 0.001* |
| CP | 0.050 | 0.020 | 0.025 | 0.021 | 0.020 | 0.017 | 0.018 | 0.017 | 0.016 | 0.015 | 0.010 | 0.008 | 0.008 | 0.006 | 0.002 |  | 0.050 |
| PB | 0.045 | 0.019 | 0.022 | 0.020 | 0.020 | 0.015 | 0.015 | 0.016 | 0.015 | 0.015 | 0.010 | 0.006 | 0.009 | 0.006 | 0.004 | 0.002 |  |
