# Supplementary material for: Landscape Genomics Provides Evidence of Ecotypic Adaptation and a Barrier to Gene Flow at Treeline for the Arctic Foundation Species Eriophorum vaginatum
Source: Front Plant Sci. 2022 Mar 24;13:860439. doi: 10.3389/fpls.2022.860439 (PMC8987161; doi:10.3389/fpls.2022.860439)
Supplement: Supplementary file 4 [file Table_4.DOCX]

**Supplementary Table S4.** Analysis of Molecular Variance (AMOVA) results for 17 *Eriophorum vaginatum* sites in north central Alaska for the neutral SNP data set.

| Source of Variation | DF | % Variation | *F*-statistic | *p*-value |
| --- | --- | --- | --- | --- |
| Between Clusters | 2 | 3.112 | 0.031 | 0.01 |
| Between Sites Within Clusters | 14 | 2.024 | 0.021 | 0.01 |
| Within Sites | 256 | 94.864 | 0.051 | 0.01 |
