# Supplementary material for: Landscape Genomics Provides Evidence of Ecotypic Adaptation and a Barrier to Gene Flow at Treeline for the Arctic Foundation Species Eriophorum vaginatum
Source: Front Plant Sci. 2022 Mar 24;13:860439. doi: 10.3389/fpls.2022.860439 (PMC8987161; doi:10.3389/fpls.2022.860439)
Supplement: Supplementary file 5 [file Table_5.DOCX]

**Supplementary Table S5.** *divMigrate* *G*_ST_ based migration values. Values by row indicate migration for each site going into sites listed by column. Migration values ≥ 0.8 are in bold.

|  | EC | NC | VM | CC | EL | NN | GO | CF | ST | TB | CH | AT | TL | AN | SG | CP | PB |
| --- | --- | --- | --- | --- | --- | --- | --- | --- | --- | --- | --- | --- | --- | --- | --- | --- | --- |
| EC |  | 0.457 | 0.408 | 0.418 | 0.425 | 0.416 | 0.409 | 0.409 | 0.344 | 0.328 | 0.313 | 0.321 | 0.300 | 0.325 | 0.345 | 0.338 | 0.371 |
| NC | 0.484 |  | 0.646 | 0.792 | 0.771 | 0.762 | 0.712 | 0.745 | 0.556 | 0.548 | 0.503 | 0.517 | 0.491 | 0.537 | 0.538 | 0.586 | 0.577 |
| VM | 0.430 | 0.685 |  | 0.660 | 0.699 | 0.718 | 0.652 | 0.670 | 0.527 | 0.511 | 0.497 | 0.477 | 0.457 | 0.474 | 0.492 | 0.531 | 0.556 |
| CC | 0.450 | 0.779 | 0.623 |  | **0.800** | **0.803** | 0.737 | 0.750 | 0.551 | 0.566 | 0.544 | 0.507 | 0.491 | 0.513 | 0.572 | 0.583 | 0.591 |
| EL | 0.454 | 0.766 | 0.656 | **0.807** |  | 0.765 | 0.733 | 0.764 | 0.563 | 0.538 | 0.524 | 0.540 | 0.499 | 0.528 | 0.516 | 0.590 | 0.588 |
| NN | 0.458 | 0.720 | 0.639 | 0.790 | 0.760 |  | **0.858** | **0.885** | 0.641 | 0.611 | 0.585 | 0.545 | 0.524 | 0.545 | 0.585 | 0.643 | 0.619 |
| GO | 0.431 | 0.653 | 0.623 | 0.733 | 0.715 | **0.816** |  | **0.932** | 0.661 | 0.645 | 0.565 | 0.569 | 0.513 | 0.555 | 0.561 | 0.626 | 0.648 |
| CF | 0.440 | 0.742 | 0.617 | 0.741 | 0.783 | **0.878** | **0.950** |  | 0.729 | 0.674 | 0.624 | 0.627 | 0.546 | 0.608 | 0.615 | 0.674 | 0.660 |
| ST | 0.376 | 0.548 | 0.471 | 0.565 | 0.570 | 0.637 | 0.659 | 0.681 |  | 0.622 | 0.603 | 0.594 | 0.573 | 0.612 | 0.647 | 0.647 | 0.628 |
| TB | 0.356 | 0.542 | 0.505 | 0.577 | 0.581 | 0.631 | 0.700 | 0.719 | 0.654 |  | 0.654 | 0.674 | 0.594 | 0.656 | 0.689 | 0.694 | 0.662 |
| CH | 0.360 | 0.516 | 0.448 | 0.538 | 0.552 | 0.572 | 0.577 | 0.657 | 0.622 | 0.641 |  | 0.795 | 0.768 | 0.771 | **0.822** | 0.772 | 0.741 |
| AT | 0.359 | 0.514 | 0.455 | 0.520 | 0.527 | 0.560 | 0.552 | 0.613 | 0.612 | 0.660 | 0.798 |  | **0.827** | **0.834** | **0.972** | **0.810** | **0.837** |
| TL | 0.333 | 0.503 | 0.432 | 0.514 | 0.523 | 0.551 | 0.541 | 0.608 | 0.634 | 0.625 | **0.860** | **0.856** |  | **0.861** | **1.000** | **0.854** | **0.814** |
| AN | 0.360 | 0.558 | 0.485 | 0.550 | 0.588 | 0.590 | 0.598 | 0.652 | 0.680 | 0.693 | **0.875** | **0.894** | **0.859** |  | **0.891** | **0.825** | **0.834** |
| SG | 0.373 | 0.544 | 0.475 | 0.581 | 0.547 | 0.598 | 0.589 | 0.631 | 0.660 | 0.652 | **0.855** | **0.932** | **0.959** | **0.884** |  | **0.987** | **0.896** |
| CP | 0.384 | 0.560 | 0.498 | 0.584 | 0.608 | 0.632 | 0.619 | 0.666 | 0.635 | 0.628 | 0.745 | 0.781 | 0.764 | 0.795 | **0.953** |  | **0.916** |
| PB | 0.402 | 0.570 | 0.503 | 0.577 | 0.575 | 0.669 | 0.648 | 0.694 | 0.661 | 0.651 | 0.746 | **0.815** | 0.733 | 0.768 | **0.895** | **0.896** |  |
