# Supplementary material for: Landscape Genomics Provides Evidence of Ecotypic Adaptation and a Barrier to Gene Flow at Treeline for the Arctic Foundation Species Eriophorum vaginatum
Source: Front Plant Sci. 2022 Mar 24;13:860439. doi: 10.3389/fpls.2022.860439 (PMC8987161; doi:10.3389/fpls.2022.860439)
Supplement: Supplementary file 6 [file Table_6.DOCX]

**Supplementary Table S6.** Values for retained RDA variables ((all except MEM1 and MEM2 obtained from the WorldClim 2.0 Bioclimatic database (Fick and Hijmans, 2017). See **Table 1** for 17 site abbreviations, iso = Isothermality, tdq = Mean temperature of driest quarter (Feb, Mar, Apr), twq = Mean temperature of warmest quarter (Jun, Jul, Aug), prd = Precipitation of driest month (Apr), prs = Precipitation seasonality.

| Site | iso | tdq (°C) | twq (°C) | prd (mm) | prs | MEM1 | MEM2 |
| --- | --- | --- | --- | --- | --- | --- | --- |
| EC | 24.89 | -13.00 | 11.37 | 6.00 | 73.46 | -1.32 | -0.72 |
| NC | 22.92 | -13.70 | 12.62 | 10.00 | 57.89 | -1.46 | -0.71 |
| VM | 22.47 | -12.98 | 11.57 | 7.00 | 59.66 | -1.46 | -0.70 |
| CC | 21.14 | -12.75 | 14.08 | 8.00 | 58.04 | -1.35 | -0.30 |
| EL | 21.27 | -11.92 | 12.18 | 8.00 | 68.07 | -1.27 | -0.10 |
| NN | 21.80 | -15.13 | 14.55 | 10.00 | 61.60 | -0.82 | 0.89 |
| GO | 19.38 | -13.57 | 12.98 | 11.00 | 59.46 | -0.10 | 1.59 |
| CF | 21.73 | -14.32 | 12.77 | 9.00 | 61.63 | 0.08 | 1.55 |
| ST | 22.33 | -15.23 | 9.38 | 7.00 | 66.91 | 0.54 | 1.27 |
| TB | 22.29 | -15.25 | 9.55 | 10.00 | 62.59 | 0.54 | 1.27 |
| CH | 21.83 | -16.03 | 8.60 | 9.00 | 62.11 | 0.76 | 0.88 |
| AT | 20.21 | -16.98 | 6.18 | 10.00 | 62.47 | 0.92 | 0.05 |
| TL | 21.73 | -19.75 | 9.93 | 7.00 | 64.45 | 1.06 | -0.50 |
| AN | 21.45 | -22.30 | 11.00 | 7.00 | 62.08 | 0.98 | -0.59 |
| SG | 21.44 | -26.58 | 10.67 | 6.00 | 60.53 | 1.00 | -1.15 |
| CP | 19.61 | -26.63 | 9.23 | 5.00 | 65.70 | 0.96 | -1.36 |
| PB | 15.96 | -27.42 | 6.60 | 4.00 | 65.10 | 0.94 | -1.38 |
