# Supplementary material for: Landscape Genomics Provides Evidence of Ecotypic Adaptation and a Barrier to Gene Flow at Treeline for the Arctic Foundation Species Eriophorum vaginatum
Source: Front Plant Sci. 2022 Mar 24;13:860439. doi: 10.3389/fpls.2022.860439 (PMC8987161; doi:10.3389/fpls.2022.860439)
Supplement: Supplementary file 7 [file Table_7.DOCX]

**Supplementary Table S7.** Annotated *E. vaginatum* candidate genes with a percentage identity match of at least 80.0 and an E-value threshold of at least 1 X 10-4 and RDA R^2^ value between 0.7 and 0.79.

| Locus ID | Gene ID | RDA R^2^ | RDA Top Predictor |
| --- | --- | --- | --- |
| 42747 | Ribonuclease H domain | 0.795 | MEM1 |
| 82292 | Acyltransferase-like protein | 0.794 | MEM1 |
| 1523776 | DEAD-box ATP-dependent RNA helicase | 0.793 | MEM1 |
| 66558 | Cysteine-rich receptor-like protein kinase 10-like | 0.791 | MEM1 |
| 43633 | Protein ENL-like | 0.780 | MEM1 |
| 74218 | AAA ATPase At3g50940-like | 0.778 | MEM1 |
| 73664 | Cellulose synthase-like protein G2-like | 0.778 | MEM1 |
| 70372 | Acyl-binding domain-containing-4 | 0.771 | tdq |
| 1963599 | Magnesium transporter NIPA2-like | 0.769 | MEM1 |
| 71022 | Dynamin-related protein 5A | 0.767 | MEM1 |
| 47232 | Zinc finger-like protein | 0.764 | MEM1 |
| 1721445 | Zinc-dependent exopeptidases superfamily protein | 0.756 | twq |
| 32318 | UPF0496 protein 4 | 0.755 | MEM1 |
| 45472 | Pentatricopeptide repeat-containing mitochondrial-like | 0.747 | tdq |
| 1405 | Actin cytoskeleton-regulatory complex PAN1 isoform X1 | 0.744 | MEM1 |
| 68114 | Flowering time control protein FCA isoform X2 | 0.725 | MEM1 |
| 32648 | Heavy metal ATPase | 0.714 | MEM1 |
| 36463 | ATP-dependent zinc metalloprotease FTSH chloroplastic | 0.713 | MEM1 |
