# Supplementary figures and images for: Landscape Genomics Provides Evidence of Ecotypic Adaptation and a Barrier to Gene Flow at Treeline for the Arctic Foundation Species Eriophorum vaginatum
Source: Front Plant Sci. 2022 Mar 24;13:860439. doi: 10.3389/fpls.2022.860439 (PMC8987161; doi:10.3389/fpls.2022.860439)

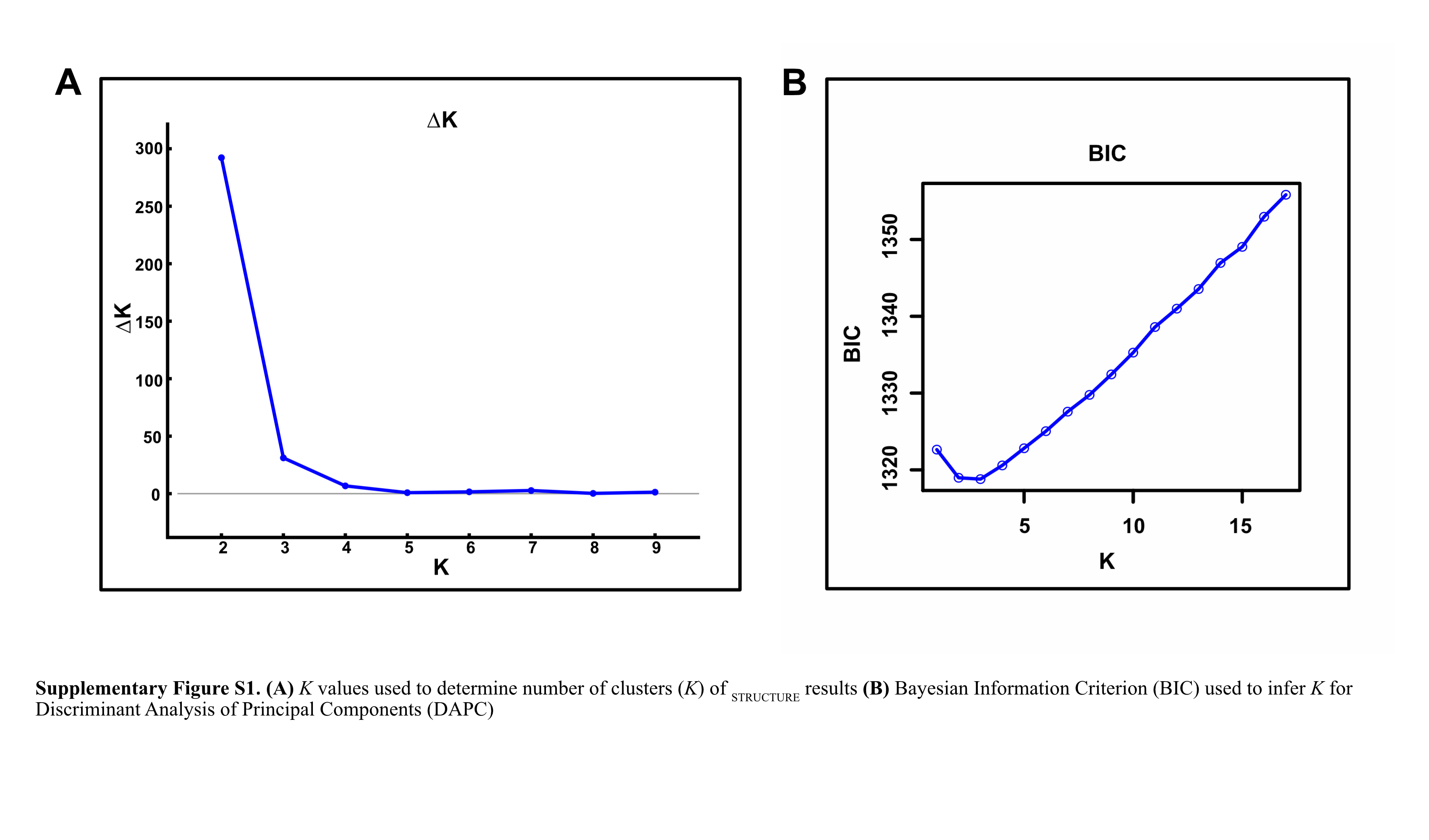

Supplement: Supplementary file 8 [file Image_1.jpg]
